# Danger signals activate a putative innate immune system during regeneration in a filamentous fungus

**DOI:** 10.1371/journal.pgen.1007390

**Published:** 2018-11-30

**Authors:** Elizabeth Medina-Castellanos, José Manuel Villalobos-Escobedo, Meritxell Riquelme, Nick D. Read, Cei Abreu-Goodger, Alfredo Herrera-Estrella

**Affiliations:** 1 Laboratorio Nacional de Genómica para la Biodiversidad-Unidad de Genómica Avanzada, Cinvestav, Libramiento Norte Carretera Irapuato-León, Irapuato, Gto, Mexico; 2 Department of Microbiology, Centro de Investigación Científica y de Educación Superior de Ensenada (CICESE), Carretera Ensenada-Tijuana No. 3918, Ensenada, Baja California, Mexico; 3 Manchester Fungal Infection Group, Division of Infection, Immunity and Respiratory Medicine, University of Manchester, Manchester, United Kingdom; University of Oxford, UNITED KINGDOM

## Abstract

The ability to respond to injury is a biological process shared by organisms of different kingdoms that can even result in complete regeneration of a part or structure that was lost. Due to their immobility, multicellular fungi are prey to various predators and are therefore constantly exposed to mechanical damage. Nevertheless, our current knowledge of how fungi respond to injury is scarce. Here we show that activation of injury responses and hyphal regeneration in the filamentous fungus *Trichoderma atroviride* relies on the detection of two danger or alarm signals. As an early response to injury, we detected a transient increase in cytosolic free calcium ([Ca^2+^]_c_) that was promoted by extracellular ATP, and which is likely regulated by a mechanism of calcium-induced calcium-release. In addition, we demonstrate that the mitogen activated protein kinase Tmk1 plays a key role in hyphal regeneration. Calcium- and Tmk1-mediated signaling cascades activated major transcriptional changes early following injury, including induction of a set of regeneration associated genes related to cell signaling, stress responses, transcription regulation, ribosome biogenesis/translation, replication and DNA repair. Interestingly, we uncovered the activation of a putative fungal innate immune response, including the involvement of HET domain genes, known to participate in programmed cell death. Our work shows that fungi and animals share danger-signals, signaling cascades, and the activation of the expression of genes related to immunity after injury, which are likely the result of convergent evolution.

## Author summary

The idea of regenerating lost body parts has always fascinated humans due to its impact on human health. Recently, the study of the response to damage has gained importance not only in regenerative medicine, but also in organ transplantation. We have established a microbial model that given its relative simplicity and ease to work with, promises to accelerate the advance of our understanding of damage responses and regeneration. We show that activation of injury responses and hyphal regeneration in a filamentous fungus relies on the detection of danger (also called alarm) signals also used by plants and animals. Finally, we discovered a set of genes that become active in response to injury, including those putatively participating in the immune response.

## Introduction

The idea of regenerating lost body parts has fascinated humans since the beginning of history [1]. Human imagination was further captured upon witnessing the extraordinary capacity of species from almost all Phyla to, upon damage, regenerate a lost part or structure [1]. Nevertheless, our understanding of the biological significance and molecular mechanisms underpinning this remarkable phenomenon and its evolution is still poor.

Multicellular organisms establish interactions with a great variety of other, potentially harmful, organisms throughout their life. Consequently, they require mechanisms to detect injury and to distinguish self- from non-self. Discrimination of self/non-self is a ubiquitous and essential function, which in animals relies on the immune system. Similarly, multicellular organisms require alarm signals known as Damage-Associated Molecular Patterns (DAMPs) to contend with a wound. In this regard, normal cellular components released into extracellular spaces, such as DNA, ATP and Ca^2+^, represent reliable signals that indicate to other cells the disruption of tissue, and trigger a response [2]. Intracellular signaling after injury involves Mitogen Activated Protein Kinases (MAPKs), as in the case of axon regeneration after spinal cord injury in vertebrates [3] and herbivory in plants [4].

Plant and animal cells have the ability to detect extracellular ATP (eATP) through recognition by specific, yet unrelated, receptors [2,5,6]. Activation of purinergic receptors or mechano-sensors triggers a transient increase in intracellular Ca^2+^ [7, 8]. In plants, eATP promotes calcium influxes after wounding [9] and large increases in eATP serve as a key “danger” signals in the inflammatory processes of zebrafish and humans [8, 10]. In animals, danger signals activate genes involved in cell signaling, stress responses, tissue patterning, cell matrix remodeling and growth [11–13]. Reactive Oxygen Species (ROS) are also considered as danger signals in plants and animals, and necessary to prevent infections during wound healing [2]. Sustained production of ROS is required for regeneration in Xenopus and zebrafish [14,15]. Furthermore, ROS is involved in the regulation of intracellular Ca^2+^ levels [16].

During tissue regeneration, a competent immune system is essential for effective wound healing [17]. Common features of innate immunity in vertebrates, invertebrate and plants include the basic chemical structure of signal molecules, signaling cascades, production of antimicrobial molecules, and transcriptional activation of defense genes [2, 18].

Fungi like other organisms have natural predators, including fungivorous nematodes and arthropods, consequently they need effective mechanisms to contend with and survive injury. When fungal hyphae are damaged, the septal pore nearest to the point of injury is sealed to prevent excessive cytoplasmic leakage. Thereafter, new hyphal tips are generated from this position, resulting in regeneration and re-initiation of growth [18].

The common soil fungus *Trichoderma atroviride* responds to mycelial injury by rapidly regenerating its hyphae and, developing asexual reproductive structures (conidia) in a NADPH oxidase (Nox) dependent manner [19]. Interestingly, application of eATP also induces conidiation [20]. Like other multicellular eukaryotes, this fungus appears to perceive eATP, through a yet unidentified receptor, which triggers activation of the MAPKs Tmk1 and Tmk3 [20]. Tmk3 is activated in a Nox1-NoxR dependent fashion, whereas Tmk1 activation is independent of Nox [20]. Mutants in either *tmk1* or *tmk3* are affected in injury-induced conidiation. Interestingly, depletion of extracellular Ca^2+^ blocked injury-induced conidiation but allowed activation of both MAPKs [20].

In addition to its role in injury-induced conidiation, Tmk3 regulates tolerance to heat shock, osmotic and oxidative stress, and cell wall integrity [21]. Unexpectedly, *tmk*3 mutants are also impaired in light-induced conidiation [21]. In contrast Δ*tmk*1 mutants, albeit of a different *T*. *atroviride* strain, hyperconidiate under standard cultivation conditions [22]. Tmk1 has also been suggested to regulate mycoparasitic activity and hyphal fusion [22, 23].

Here we provide new mechanistic insights into the activation of a regeneration program, consisting of genes involved in cell signaling, stress responses, transcription, ribosome biogenesis/translation, DNA replication, growth, and defense through Ca^2+^/MAPK-dependent signaling pathways. Finally, we uncover the activation of genes of a putative fungal innate immune response involving genes previously known to participate in heterokaryon incompatibility [24].

## Results

### Injury induces a transient increase in [Ca^2+^]_c_ that is required for transcriptional regulation of hyphal regeneration

**Fig 1:**
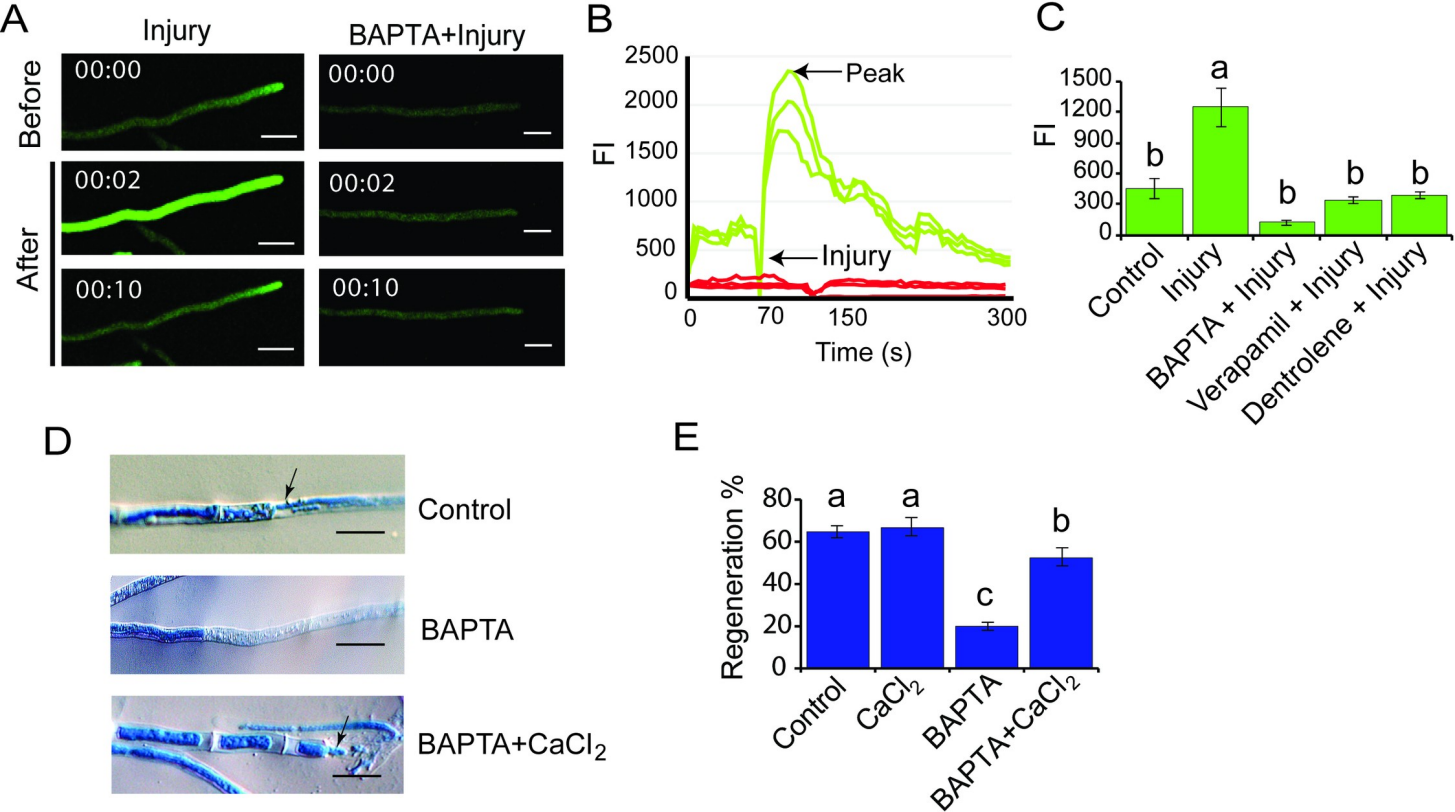
Role of Ca^2+^ in the early response to injury. **A.** Live-cell imaging. The *T*. *atroviride* WT strain carrying the sensor GCamP6 was damaged with a scalpel, or exposed to BAPTA and then damaged. Images were obtained using time-lapse confocal microscopy. Scale bar = 10 μM. Time shown in seconds. **B.** The graph shows the Fluorescence Intensity (FI) per hypha of the WT strain carrying the sensor GCamP6 for three representative hyphae during injury (green line) and treated with BAPTA and then injured (red lines) for three representative hyphae. **C**. Effect of calcium channels inhibitors on [Ca^2+^]_c_. The graph shows the mean maximum change of FI approximately 2–6 sec after injury. In each case the WT strain was treated with the indicated Ca^2+^ inhibitor before injury. Four independent experiments were performed for each treatment. **D.** Microscopic changes. One hour after injury hyphae were stained with lactophenol cotton blue and examined by light microscopy. Treatments were: Control: normal regeneration response of injured hyphae; BAPTA: hyphae did not regenerate after being exposed to BAPTA for 15 min and then damaged; BAPTA+CaCl_2_: partial restoration of hyphal regeneration following exposure to BAPTA for 15 min and then addition of 0.34 mM CaCl_2_. Arrows point to the new hyphae. Scale bar = 10μM. **E**. Regeneration capacity. The graph shows the percentage of hyphae that regenerate after each treatment. Three independent experiments were performed for each treatment, counting 50 hyphae in each case. **C, E**. Bars represent the mean ± s.e.m. A one-way ANOVA test, followed by Tukey Honest Significant Differences was used. Different letters indicate significant differences (*P <* 0.05).

Considering the relevant role of calcium in regeneration in other organisms, we evaluated calcium signatures at wound sites in *T*. *atroviride*. Live-imaging analysis of wounded cells, expressing the Ca^2+^ sensor GCamP6, revealed a transient spike of cytosolic free calcium ([Ca^2+^]_c_**)** immediately after injury (Fig 1A, S1 and S2 Movies). To determine if extracellular calcium was involved in this response, we applied the calcium-chelating agent BAPTA. Injury promoted a transient elevation of [Ca^2+^]_c_, while treatment with BAPTA prior to injury completely suppressed this response (Fig 1A). The injury-induced calcium signature showed a very rapid and strong peak of increase in [Ca^2+^]_c_ that decreased over time (Fig 1B). In contrast, in presence of BAPTA, the [Ca^2+^]_c_ was not altered upon injury (Fig 1B). The decrease in fluorescence observed in the graphs just before injury (indicated by an arrow) is an unavoidable artifact due to the introduction of the scalpel used to cause the injury, which blocks light. To test if the observed response involved uptake of extracellular calcium and/or release of calcium from intracellular pools, we applied the calcium release inhibitors verapamil, which blocks L-type calcium channels in the plasma membrane, and dantrolene that inhibits Ca^2+^-induced Ca^2+^release from the sarcoplasmic reticulum pool by targeting the ryanodine receptor [25]. Both compounds significantly reduced [Ca^2+^]_c_ increases due to injury (Fig 1C), consistent with the participation of a calcium-induced calcium release system. Next, we analyzed the role of calcium in the regeneration process. Application of BAPTA to the fungal mycelium prior to injury reduced regenerating hyphae to 20%, as compared to 64% observed in an untreated control (Fig 1D and 1E). Addition of extracellular calcium to hyphae previously exposed to BAPTA partially restored regeneration upon damage, as evidenced by the formation of thin new hyphae as a result of tip growth re-initiation (Fig 1D**)** and an increase in regeneration (52%; Fig 1E**).**

**Fig 2:**
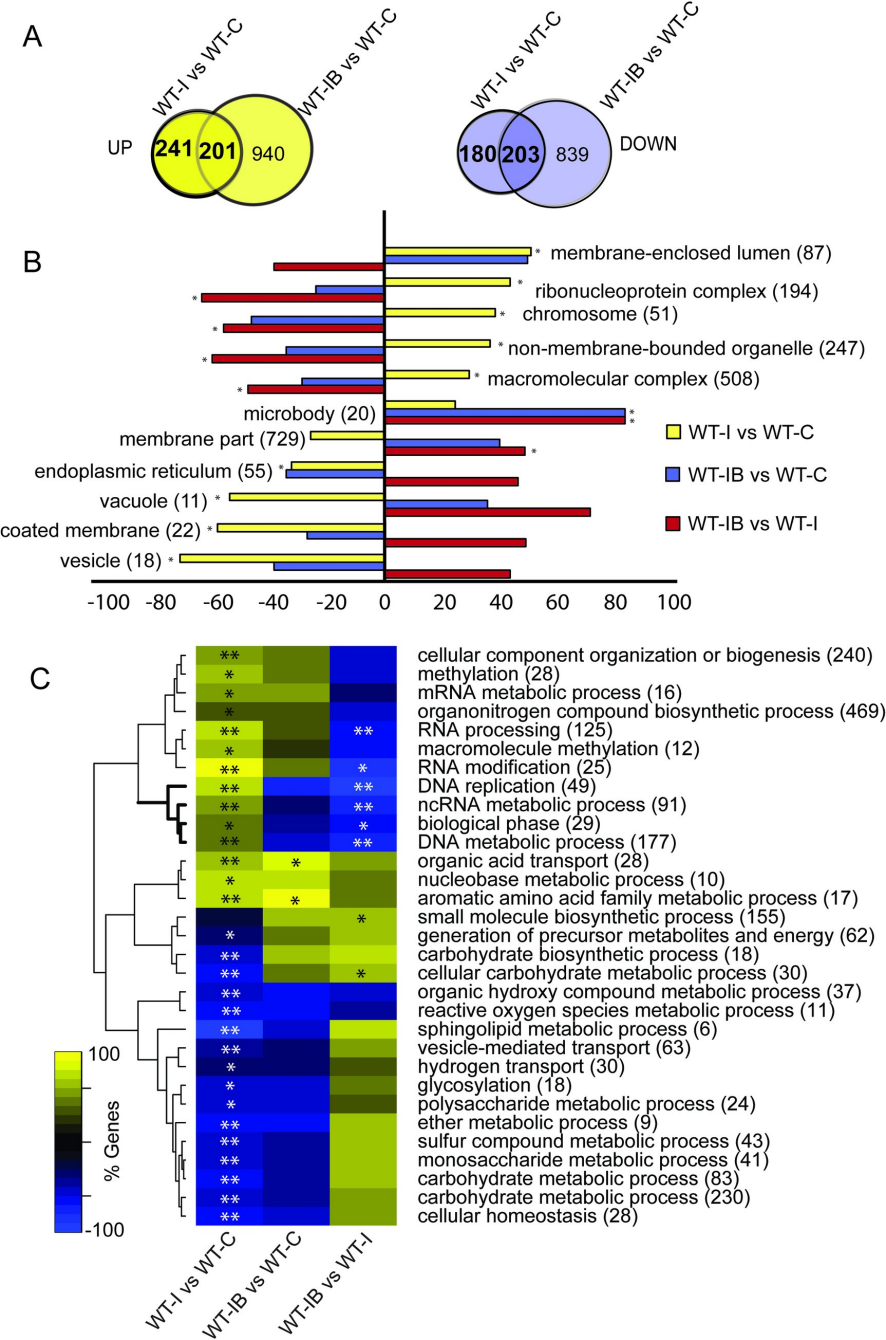
Calcium is essential for the transcriptional response to injury. **A.** Venn diagrams show the overlap of the induced (yellow) and repressed (purple) genes of the WT strain in response to injury (WT-I) and in response to injury after BAPTA treatment (WT-IB), as compared to an untreated control (WT-C). **B.** Enrichment analysis using Cellular Component Gene Ontology (GO) terms, showing the percentage of induced or repressed genes belonging to each category (FDR <0.05*). **C.** Clustering of significantly enriched Biological Process GO terms, showing the percentage of induced or repressed genes belonging to each category (FDR <0.01**; FDR <0.05*). In **B** and **C,** the sign indicates the direction of change, positive/negative being higher/lower in injury than in the control. The number in parenthesis after each GO term indicates the total number of genes in that category.

We then compared the transcriptional profile of the fungus in response to Injury in the presence (IB) and absence (I) of BAPTA. We identified a total of 421 Calcium-Dependent Injury Genes (CDIGs), responsive only in the absence of BAPTA; of which 241 were up-regulated and 180 down-regulated. In addition, we identified 404 Calcium-Independent Injury-Genes (CIIGs), responsive even in the presence of BAPTA; 201 of which were up-regulated and 203 down-regulated (Fig 2A, S1–S3 Datasets). Upon injury, the fungus up-regulates genes associated to the following cellular components: chromosomes, lumen enclosed by membrane, ribonucleoprotein complexes, intracellular non-membrane-bound organelles (associated with the cytoskeleton) and macromolecular complexes (Fig 2B, WT-I vs WT-C, S4 Dataset). BAPTA chelation of calcium blocked genes that were associated with chromosomes, and non-membrane-bounded organelles, but induced genes related to microbodies (vacuoles), apparently in an injury independent fashion (Fig 2B, WT-IB vs WT-C & WT-IB vs WT-I). Moreover, injury caused an increase in genes belonging to the biological processes of RNA processing, translation, DNA metabolic processes and replication (Fig 2C; WT-I vs WT-C). Remarkably, upon injury, but in the absence of extracellular calcium, there was no significant enrichment in these processes (Fig 2C, WT-IB vs WT-C).

### eATP is a primary danger signal that induces Ca^2+^ influx and triggers hyphal regeneration

**Fig 3:**
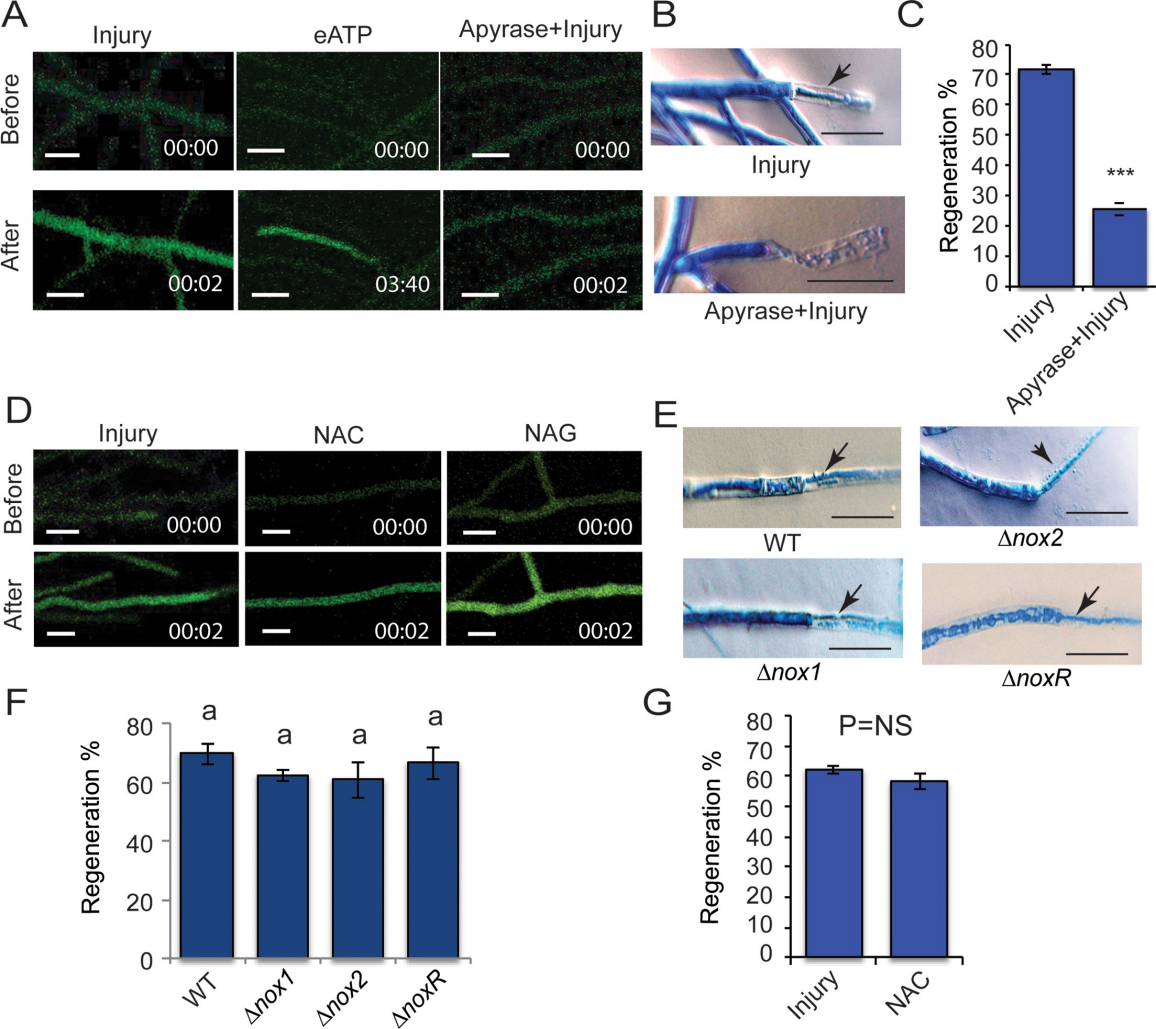
Role of eATP and ROS in Ca^2+^ influx and regeneration. **A & D.** Live-cell imaging of the *T*. *atroviride* WT strain carrying pEM12 treated with 100 μM ATP (eATP) or apyrase and then injured (**A**) or 30 mM NAC or NAG and then damaged (**D**), the WT strain subjected only to injury was used as a control. Images were obtained using time-lapse confocal microscopy whilst applying the treatment. Scale bar = 10 μM. Time shown in minutes. **B & E.** Microscopic changes observed after injury. The photographs in **B** show the response of the WT strain upon injury or treatment with apyrase for 15 min and then damaged. The images in **E** show the response of the *Δnox1*, *Δnox2* and *ΔnoxR* mutants upon damage. Hyphae were stained with lactophenol cotton blue and examined by light microscopy. Arrows point to the new regenerating hyphae. Scale bar = 10 μM. **C.** The graph shows the percentage of hyphae of the wild type strain that regenerate upon injury or apyrase treatment prior to injury. **G.** The graph shows the percentage of hyphae of the WT strain that regenerate upon injury and those that regenerate when injured after exposure to 30 mM NAC (NAC). **C & G.** Bars represent the mean ± s.e.m. A t-test was performed, with a significant (*P <* 0.001***) or non-significant (*P* = NS) difference. **F.** The graph shows the percentage of hyphae in the *Δnox1*, *Δnox2* and *ΔnoxR* mutants that regenerate upon injury. Bars represent the mean ± s.e.m. A one-way ANOVA was used. There was no significant difference between treatments (*P* < 0.05) as indicated by *P* = NS. **C, F & G.** Three independent experiments were performed for each treatment, counting 50 hyphae in each case. There was no difference between treatments (*P* < 0.05) as indicated by *P* = NS.

We had previously suggested that eATP could function as a DAMP [20]. To determine if indeed eATP serves as a DAMP that triggers Ca^2+^ influxes in *T*. *atroviride* after wounding, we evaluated Ca^2+^ dynamics and regeneration, upon addition of eATP or apyrase, an enzyme that hydrolyses ATP to AMP. Addition of eATP without injury provoked an increase of [Ca^2+^]_c_ (Fig 3A). However, when the mycelium was pre-treated with apyrase, the fluorescence signal after injury was abolished, as compared to an untreated (no apyrase) control (Fig 3A**).** Furthermore, damaged hyphae treated with apyrase showed a strongly reduced regeneration capacity (Fig 3B): only 26% of hyphae regenerated (Fig 3C). We also evaluated the transcriptional changes that occur when adding eATP; remarkably, as upon injury, we found that gene expression associated with DNA replication, the cell cycle, RNA biosynthetic processes and organic acid transport was induced (S1 Fig, S5 Dataset). Thus, ATP released from damaged cells promotes regeneration, likely by promoting calcium influxes.

Similarly, we evaluated if ROS is necessary for triggering Ca^2+^ influx and hyphal regeneration. For this purpose, we evaluated changes in [Ca^2+^]_c_ upon injury in presence of the antioxidant N-acetyl-Cysteine (NAC) and its analog N-acetyl-glycine (NAG), as control. After injury, a spike of [Ca^2+^]_c_ was observed even in the absence of ROS (NAC treatment) (Fig 3D**),** suggesting that ROS are not required to trigger Ca^2+^ influxes and possibly regeneration. To determine if NOX-dependent ROS production plays a role in hyphal regeneration, we performed regeneration assays using the *Δnox1*, *Δnox2* and *ΔnoxR* mutants. In all cases emergence of new hypha from the cell adjacent to the broken one was observed (Fig 3E). All mutants showed the same capacity to regenerate observed in the WT strain (Fig 3F). To explore if ROS regardless of its source played a role in regeneration, we applied a NAC treatment prior to injury, observing no difference in the percentage of regeneration compared with the untreated control (Fig 3F). These results are consistent with eATP acting as a DAMP (a form of “danger signal”), that activates a Ca^2+^ influx which is necessary for hyphal regeneration. In contrast, ROS are not involved in causing the transient elevation in [Ca^2+^]_c_ nor in the regeneration process.

### Tmk1 is essential for hyphal regeneration

**Fig 4:**
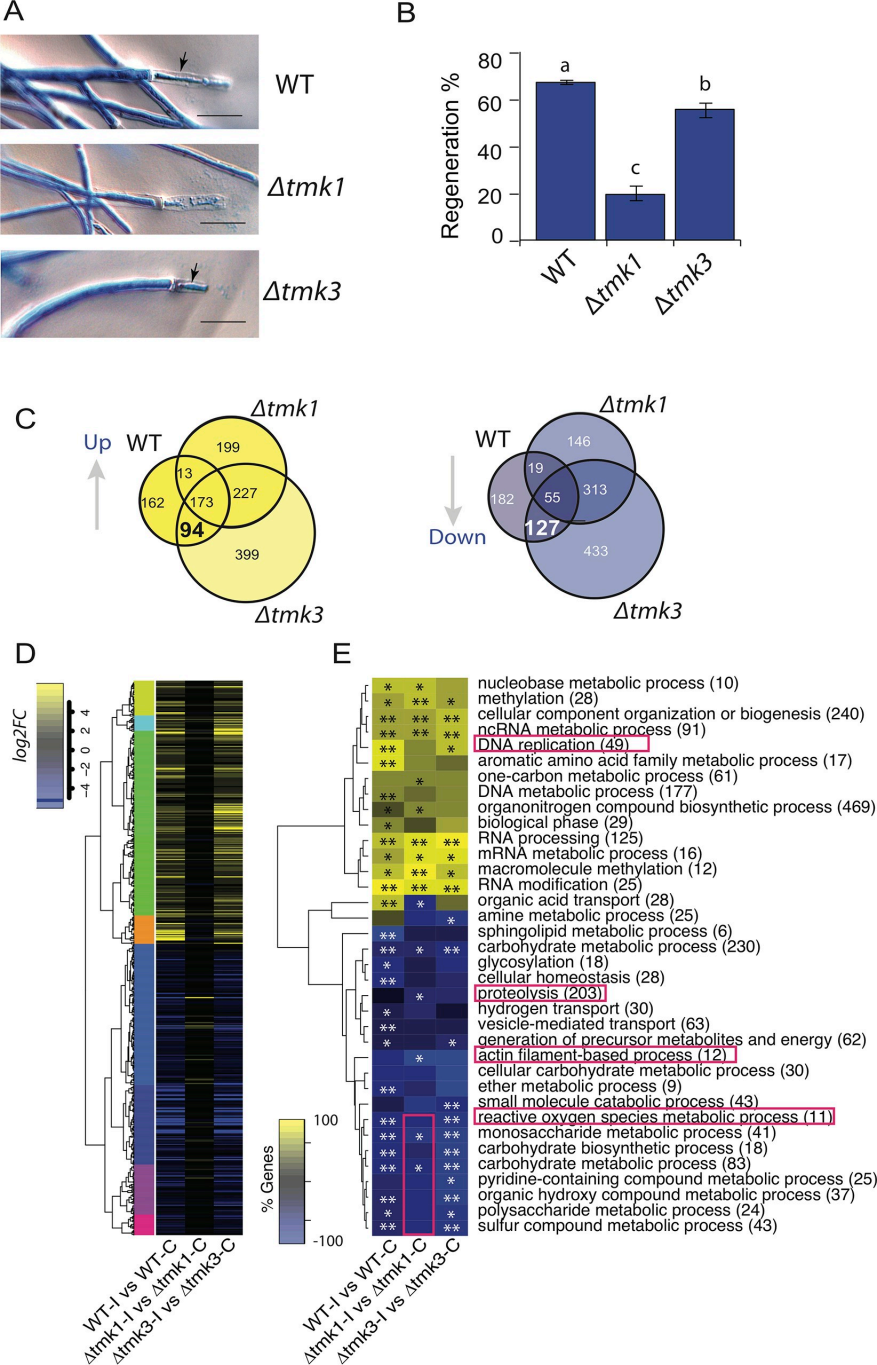
Tmk1 signaling is required for hyphal regeneration and correct transcriptional response. **A.** Microscopic changes observed 1 h after injury in the WT, Δ*tmk*1 and Δ*tmk*3. Hyphae were stained with lactophenol cotton blue and examined by light microscopy. Arrows point to the new hyphae. **B**. The graph shows the percentage of hyphae that regenerate upon injury in each strain. Three independent experiments were performed for each treatment, counting 50 hyphae in each case. Bars represent the mean ± s.e.m. A one-way ANOVA test, followed by Tukey Honest Significant Differences was used. Different letters indicate significant differences (*P <* 0.05). **C.** Venn diagrams showing the overlap between up-regulated and down-regulated genes that respond to injury in the WT, Δ*tmk*1 and Δ*tmk*3 strains. **D.** Heat map showing the expression profile of the Injury-Responsive Tmk1 dependent (IRK1) genes and their behavior following injury in each strain (FDR < 0.05; Fold-change > 1). **E.** Heat map with enriched GO biological process terms, showing the percentage of genes belonging to each category (FDR <0.01**; FDR <0.05*). The number in parenthesis after each GO term indicates the total number of genes in each category.

To determine if MAPK signal transduction pathways are involved in the regeneration process, we used gene replacements mutants of the MAPK encoding genes *tmk*1 and *tmk*3. Hyphal regeneration in the WT, Δ*tmk*1 and Δ*tmk*3 strains was analyzed after damage with a scalpel. The regenerative capacity of the Δ*tmk*1 mutant was drastically affected, since in many cases we did not observe the emergence of new hyphae (Fig 4A**)**. On average, only 20% of the hyphae showed regeneration in the absence of Tmk1, compared with 68% in the wild type strain (Fig 4B). In contrast, the Δ*tmk*3 mutant exhibited only a slight decrease in regenerative capacity, with 56% of the hyphae regenerating (Fig 4B**)**.

To identify injury-responsive genes linked to MAPKs, we performed a transcriptional analysis using RNA extracted from the *Δtmk1* and *Δtmk3* mutants upon injury and compared their transcriptional profiles with that of the WT strain. We identified a set of Injury-Responsive Tmk1 dependent (IRK1) genes, whose expression changes upon injury in the WT and Δ*tmk*3 strains (94 up-regulated and 127 down-regulated) were no longer observed in the Δ*tmk*1 mutant, which shows strongly reduced regeneration (Fig 4C, S6 & S7 Datasets). These analyses clearly showed that most clusters of genes differentially expressed in both the WT and Δ*tmk*3 strains remained nearly unresponsive in the Δ*tmk*1 mutant **(**Fig 4D**).** The IRK1 genes included key elements of cell signaling, DNA replication, and DNA metabolic processes (S8 Dataset). The pattern of expression of the IRK1 genes in the Δ*tmk*3 mutant was similar to that observed for the wild type strain at early stages of the response to injury. Nevertheless, we detected 13 up-regulated and 19 down-regulated genes that did not respond to injury in the Δ*tmk*3 mutant but responded to the stimulus both in the WT and Δ*tmk*1 strains (Fig 4C). Given that the latter strains can proceed into conidiation, this set of Injury-Responsive Tmk3 dependent (IRK3) genes might represent a set involved in the onset of conidiation (S8 Dataset).

A Gene Ontology analysis of the transcriptional response to injury in each strain suggests that the functional response in the *tmk*3 mutant is quite similar to that of the WT (Fig 4E). Although cellular component organization and RNA processing categories responded similarly in all three strains, the *tmk*1 mutant did not show a significant up-regulation of genes involved in DNA replication nor down-regulation of several metabolic processes, including that of reactive oxygen species (Fig 4E). On the other hand, genes encoding proteins associated with actin filaments and proteolysis, likely required for regeneration, were only down-regulated in Δ*tmk*1 (Fig 4E).

### Calcium signaling and the Tmk1 pathway drive expression of regeneration genes

**Fig 5:**
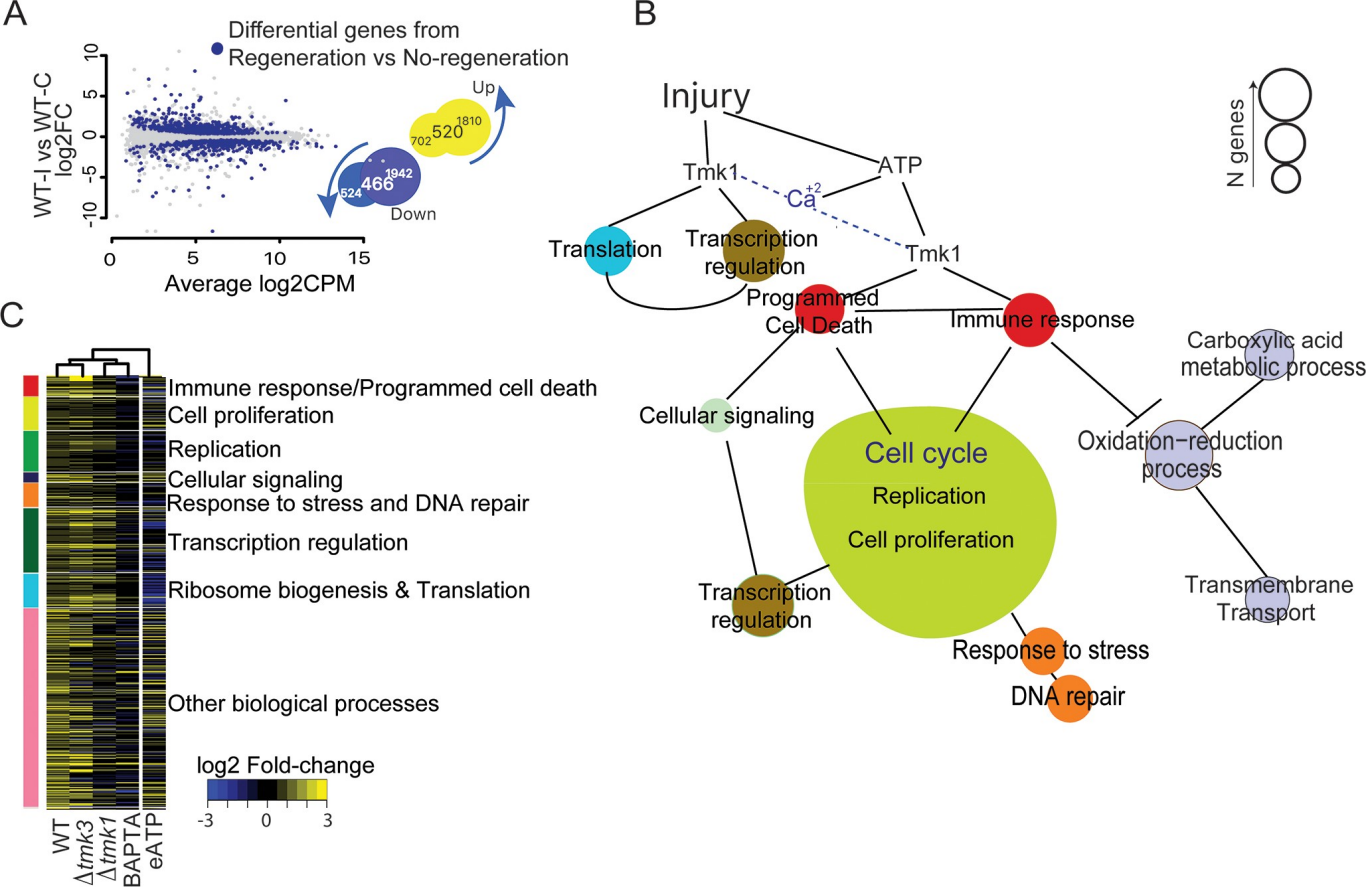
Functional analysis of Regeneration Gene Set. **A.** The plot highlights the differentially expressed genes from the *“Regeneration vs No-Regeneration”* comparison (blue dots) on the results of the WT-Injury vs WT-Control comparison. The Venn diagrams show the intersections between the two comparisons. **B.** Model based on manual inspection of regeneration genes with enriched Gene Ontology terms. Circle sizes are proportional to the number of genes contained in each category. **C.** Heatmap of the regeneration genes’ fold-change after injury in the different mutants and pharmacological treatments. Vertical color bar groups genes by GO term, colored as in **B**. Hierarchical clustering of columns was determined using the *hclust* function of the stats package in R, and the distance measured using a Pearson correlation and "complete" as the clustering method.

Until now, two of our experimental conditions led to an impaired regeneration capacity after injury: Δ*tmk*1 and WT treated with the Ca^2+^ chelator, BAPTA. We hypothesized that these facts could be used to define genes required for regeneration by comparing gene expression profiles between regenerating and non-regenerating conditions/strains: (WT injury, Δ*tmk*3 injury) versus *(*Δ*tmk*1 injury, BAPTA injury). To make sure that the resulting genes respond to injury in the WT, we then compared them with those differentially expressed in the comparison WT injury vs WT control (Fig 5A). The intersection of both groups represents what we define as the “Regeneration Associated Gene Set” (RAGS), which expression is associated with regeneration, constituted by 520 up-regulated and 466 down-regulated genes (Fig 5A). Based on Gene Ontology, the induced component of the RAGS was enriched in genes involved in DNA and RNA metabolic processes, cellular responses to stress, the cell cycle, and ribosome biogenesis, among others (S2 Fig). However, no significant functional enrichment was found for the down-regulated genes.

Closer inspection and manual annotation allowed us to classify the induced set of genes into 7 functional categories (Fig 5B, S9 Dataset). Six of the RAGS categories are involved in processes clearly associated with regeneration in many other organisms: cell proliferation (35 genes), replication (43 genes), cellular signaling (10 genes), response to stress/DNA repair (25 genes), transcription regulation (69 genes), and ribosome biogenesis/translation (36 genes). Interestingly, the seventh group (22 genes) is mostly constituted by genes related to programmed cell death and genes with a fungus-specific HET domain. Upon manual inspection of the down-regulated RGS, we found a large number of genes involved in oxido-reduction processes (S10 Dataset).

As expected, when looking at the individual treatments/strains, we observed that the RAGS did not respond to injury or showed a strongly diminished response in the Δ*tmk*1 mutant and upon BAPTA treatment. Instead they followed similar patterns of expression to those of the Δ*tmk*3 and WT strains upon injury, and after addition of ATP (Fig 5C). Additionally, we observed that application of eATP mostly mimicked the response of the genes provoked by injury in the WT (Fig 5C). Interestingly, the genes of the ribosome biogenesis/translation and transcription regulation categories, which are up-regulated upon injury of the WT, are down-regulated upon treatment with eATP. These observations indicate that eATP is not sufficient to trigger a full regeneration response (Fig 5B).

**Fig 6:**
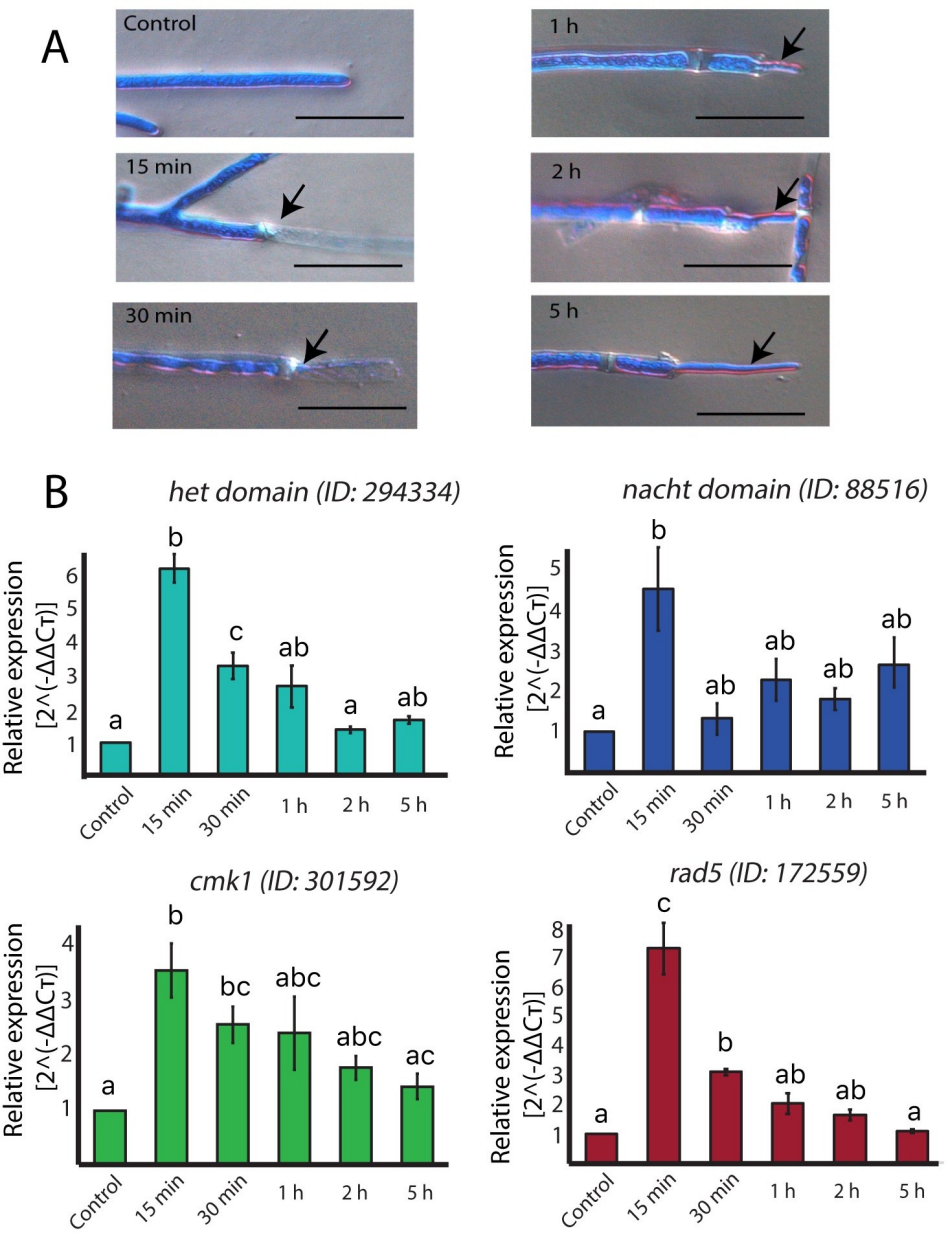
The expression of RGS correlates with the initial stages of regeneration. **A.** Microscopic changes observed after injury. Hyphae were stained with lactophenol cotton blue and examined by light microscopy. Regenerating hyphae were photographed 15, 30 minutes, and 1, 2, and 5 hours after injury. Scale bar = 10 μm. Images are representative of the morphological stage of the hyphal population at the indicated time. **B.** Relative mRNA expression of a Het domain, a Nacht domain encoding genes, and the *rad*5, and *cmk*1 genes. The expression is relative to DNA polymerase encoding gene (Id. 53190), which expression does not vary under the tested conditions. Graphs show the results of four biological and three technical replicates. Error bars represent ± s.e.m. A one-way ANOVA test, followed by Tukey test were used to determine significant differences, indicated by different letters (P < 0.05).

To better understand the dynamics of expression of the RAGS we selected four genes belonging to three different categories, namely cell proliferation (*rad*5, Id. 172559), cell signaling (*cmk*1, Id. 301592), and programmed cell death (Het domain, Id. 294334; Nacht domain, Id. 88516) for quantitative gene expression analyses. As expected, we observed a strong increase in the level of all four transcripts early after injury, reaching their maximum at 15 min, when the new regenerating hyphae become barely visible (Fig 6). The expression of the genes clearly decreases by 30 min, when the new hyphae have already emerged (Fig 6). The level of expression of all four genes then starts dropping slowly, and 5 h after injury, when most (90%) regenerated hyphae were completely evident (Fig 6A), they reached the level observed in the control.

## Discussion

We have previously shown that *T*. *atroviride* responds to mycelial injury by rapidly regenerating its hyphae and, developing conidia in a Nox-dependent manner [19]. We had also shown that eATP induces conidiation and triggers activation of Tmk1 and Tmk3, and that the latter is activated in a Nox1-NoxR dependent fashion [20]. Further, mutants in either *tmk1* or *tmk3* were affected in injury-induced conidiation, which is the final outcome of the process [20]. Intriguingly, depletion of extracellular Ca^2+^ blocked injury induced conidiation but allowed activation of both MAPKs [20]. In this regard, Ca^2+^ released from a damaged cell may be detected by neighboring cells as a signal molecule or serve as a second messenger liberated from intracellular pools and/or be transported across the plasma membrane upon detection of DAMPs. However, it was unclear whether the regeneration and conidiation processes were mechanistically linked and how all these elements were interconnected to regulate the response to damage.

In this regard, two of the earliest signaling events after wounding in animals and plants are the activation of MAPKs and Ca^2+^ influxes [2]. MAPKs are also involved in the early stages of regeneration in hydra [26] and planaria [27]. Likewise, here we show that the MAPK Tmk1 is involved in hyphal regeneration control. Furthermore, [Ca^2+^]_c_ increases are necessary for sealing the disrupted plasma membrane [28], and Ca^2+^ signaling is essential to activate defense responses in plants and the immune system in mammals [29, 30]. Similarly, we detected a transient increase in [Ca^2+^]_c_ seconds after damage, which was promoted by eATP, as one of the earliest events of the response to injury with evidence that the increase in [Ca^2+^]_c_ is regulated by extracellular Ca^2+^ involving a mechanism known as Ca^2+^-induced Ca^2+^-release [31]. Interestingly, in filamentous fungi, deletion of Cch1 or Mid1, which are components of a mechanosensing Ca^2+^-channel complex, results in diminished thigmotropic responses, and failure to establish cell polarity [32]. A comparable mechanism of mechanosensing is used by mammals during immune cell activation [33, 34]. A direct link between the increase in [Ca^2+^]_c_ and the control of transcription, was revealed by the up-regulation of the Ca^2+^/calmodulin dependent kinase CAMK1, and the transcriptional factor CRZA in response to injury. In planaria and hepatic stem cells calmodulin encoding genes are induced by wounding [13, 35], and intimately linked with the signals activating the innate immune system [36]. Moreover, we found that increasing [Ca^2+^]_c_ and eATP are necessary for the formation of new, regenerated hyphal tips and re-initiation of mycelial growth upon injury. In this regard, it has been established that actin cytoskeleton rearrangements are Ca^2+^-dependent and determine the site of tip growth and polarized cell movement [32, 34]. In plants and animals, ROS production is required during healing and regeneration [14, 15, 37]. In contrast, it appears that ROS are not required for hyphal regeneration, although they could participate as signal molecules in the early response inducing genes involved in injury-induced conidiation [18, 19]. This is consistent with the fact that Tmk3 is activated by ROS, both necessary for injury-induced conidiation but not regeneration [20]. Thus, as in plants and animals, signaling by eATP, Ca^2+^ and MAPKs appears to be essential for regeneration in filamentous fungi.

We further show that the Ca^2+^ and Tmk1 signaling pathways, appear to control the expression of thousands of genes. However, we defined a Regeneration Associated Gene Set (RAGS) in which these two signaling pathways converge. The RAGS could be involved in either the metabolic changes required to produce a new hypha and reinitiate growth (regeneration), respond to mechanical stress, and/or defense. Six different processes that could clearly be linked to regeneration are strongly represented within the RAGS. Some of the individual genes found within the RAGS encode proteins involved in cell cycle regulation and two components of the condensin complex, whose participation in regeneration processes has been documented [38, 39, 40]. It is noteworthy that we also found genes, such as *ssu*72, required for replication initiation and previously shown to participate in the control of cell cycle progression in mice in response to liver damage [41]. We also found DNA replication licensing factors (*mcm* genes), which are up-regulated during regeneration in planaria, mice and axolotl [42–44], and have been shown to play a key role in cell proliferation [45].

We defined a seventh group within the RAGS, which contains eight genes encoding HET domain proteins. In this regard, fungal hyphae from different individuals can fuse, resulting in the coexistence of genetically different nuclei in a common cytoplasm (heterokaryon). The fate of the fused cell is determined by HET domain proteins through allorecognition processes, in which heterokaryons resulting from the fusion undergo a type of programmed cell death [24, 46]. In addition, we found two induced homeodomain transcription factors, annotated as potential mating type factors. In *Neurospora crassa*, some HET domain proteins interact with mating factors to carry out the heterokaryon incompatibility process. Allorecognition processes allow the distinction of self from non-self in cells and tissues, and participate in processes, ranging from tissue transplant fusion to immune defense, across the tree of life [47].

Remarkably, in addition to HET domain protein encoding genes, we found 14 genes which participate in either cell death or the innate immune system in animals. Among them a caspase, a putative phosphatidylserine-specific receptor, a PITSLRE protein kinase, and the activation of apoptosis signal-regulating kinase 1, all of which play major roles in apoptosis [48–50]. Other interesting genes within this group were a 3–5 exoribonuclease *csl*4, a probable GMP synthase, and a Ca^2+^-independent phospholipase A2, which are key elements of the innate immune response in animals [51–53].

Fungi, like all organisms, are potential hosts for microbial pathogens and have developed defense systems against competitors and pathogens. Programmed cell death and non-self-recognition systems are considered an important strategy to contend with infections in fungi, plants and animals [16]. Furthermore, heterokaryon incompatibility has been shown to prevent various forms of somatic parasitism, and to reduce the risk of transmission of infectious cytoplasmic elements and mycoviruses [54–56]. This set of HET domain proteins together with two DEAD/H-box helicases present in our RAGS, and which have been implicated in cytosolic DNA sensing [57], could play a major role in detecting damaged or invading DNA molecules. Furthermore, in plants and animals, the innate immune response relies on specific proteins, the pattern-recognition receptors (PRRs), which detect conserved pathogen-associated molecular patterns and “danger” signals [2, 58]. The domain architecture of HET proteins is similar to that of both plant and animal cytosolic PRRs, suggesting that similar modes of activation occur even if primary sequences and downstream functions are diverse [52, 59]. These incompatibility genes are extremely polymorphic and show signatures of diversifying selection [52, 60]. Moreover, recent biochemical evidence showed that fungal NLR-like proteins function similarly to NLR immune receptors in plants and animals, concluding that NLRs are major contributors to innate immunity in three kingdoms, including fungi [59]. Consequently, HET proteins may be involved in protecting the fungus from pathogens, invading DNA/RNA molecules, and serve as damaged self-recognition system, as components of an innate immune system.

Based on these observations, we propose that a filamentous fungal innate immune response process promotes regeneration and we designate this set of 22 genes as the innate immunity group. The importance of the activation of the immune system in regeneration in organisms such as zebrafish and hydra has previously been documented [61, 62]. Thus, we postulate that there is a cellular Boolean system that determines entry into cellular proliferation, mediated by different genes involved in perception of exogenous and/or damaged genetic material, which lead to cell death if the cell/tissue damage is too extensive or caused by pathogenic organisms, since initiating DNA replication would compromise genome integrity. However, if this were not the case, rapid communication with the DNA repair and replication systems would take place and regeneration would be promoted.

According to Sanchez-Alvarado [1], the molecular cascades associated with regeneration may have appeared first and foremost as a way to asexually propagate species, and that such cascades may have been co-opted by many organisms to cope with injury. Here we show that filamentous fungi, which have the capacity to reproduce by fragmentation, share many elements thought to be exclusively used by animals for regeneration. Furthermore, although speculative at this stage, our data suggest that in fungi an innate immune system is involved in hyphal regeneration, with the participation of HET domain proteins, previously thought to exclusively trigger programmed cell death [24]. In this regard, although the domain architecture and mechanistic functioning of NLR proteins are strikingly similar, their evolution in the different kingdoms of life is thought to be convergent [63, 64]. Thus, our findings indicate that the signaling pathways involved in regeneration across kingdoms were likely co-opted to aid this process after multicellularity evolved.

## Materials and methods

### Strains and culture conditions

*Trichoderma atroviride* IMI 206040 was used as the wild type strain (WT). The *Δtmk1* and Δ*tmk*3 mutants have been described previously [20], as have the Δ*nox*1, Δ*nox*2, and Δ*nox*R mutants [19]. The *T*. *atroviride* strain carrying the Ca^2+^ sensor GCamP6 [65] (Calmodulin::GFP), was obtained by transformation with the plasmid pEM12 (see below). All strains were propagated on potato dextrose agar.

### Plasmid construction

To generate pEM12, the sequence of GCamP6 (Calmodulin::GFP) was obtained from plasmid pSK379 [66] and amplified by PCR using the primers GCaMP6-EcoRI-FW and GCaMP6-SalI-RV. *T*. *atroviride* was then transformed with pEM12, as previously described [67], and subjected to five passes through monosporic culture. All oligonucleotides used are indicated in S1 Table.

### Analysis of [Ca^2+^]_c_ dynamics following mycelial injury

Colonies of *T*. *atroviride* expressing the Ca^2+^-sensor GCamP6::GFP (pEM12) were grown on Vogel’s minimal medium (VMM) or Potato Dextrose Broth in 0.5% agar and incubated for 36 h on glass slides (Corning). Hyphae were damaged approximately 80 μm behind the tips of leading hyphae, using a scalpel. They were visualized using a confocal laser scanning microscope (CLSM) Olympus FluoView FV1000 (Olympus, Japan) fitted with an argon/2 ion laser (EGFP: excitation, 488 nm; emission, 510 nm). A 60 X Plan oil-immersion objective (1.42 N.A.) was used for image acquisition. Image projections consisting of stacks of images were captured at 5 min intervals and converted into movies using confocal FluoView FV1000 (Olympus Corp.) software.

To analyze Ca^2+^ fluxes during cell damage, we used the following Ca^2+^-modulators: 5 mM verapamil, 100 μM dantrolene, and 10 mM of cell impermeant 1,2-bis-(o-aminophenoxy)-ethane-N,N,N’,N’-tetraacetic acid) tetrapotassium Salt (BAPTA) (Life Technologies). The mycelium was incubated with these modulators for 15 minutes. The colonies were then damaged with a scalpel and visualized with a CLSM. To analyze the role of eATP in the promotion of Ca^2+^ fluxes, we treated the mycelium for 15 min with 2 units of apyrase (Sigma), an enzyme that hydrolyzes ATP, before damaging the cells. Confocal imaging was performed immediately following injury or addition of 100μM eATP. To analyze the role of ROS, we exposed the WT strain to 30 mM N-acetyl-cysteine (NAC) or 30 mM N-acetyl-glycine (NAG) for 15 min before damage. Untreated colonies without treatment were used as controls. The fluorescence intensity was quantified per hypha using Image J software. The mean change in fluorescence measurements were made in a 50 μm x 30 μm region of interest drawn over 10 hyphae approximately 80 μm back from their tips. R packages were used for statistical analysis.

### Regeneration assay

Colonies of *T*. *atroviride* WT, *Δtmk*1, *Δtmk*3, Δ*nox*1, Δ*nox*2, and Δ*nox*R mutants were grown in half strength PDB supplemented with 1% agarose for visualization of isolated regenerating hyphae as described above and incubated for 48 h at 27°C. The wild type strain was exposed to the Ca^2+^ modulators or apyrase (Sigma), as described above. Mycelia of the different strains/treatments were then damaged with a scalpel and incubated for 5 h. Finally, the mycelium was stained with lactophenol cotton blue for 10 min. Mycelia were observed on a Leica DM6000-B microscope fitted with a 40x objective HCX PL Fluotar (0.75 N.A.) and photographed with a Leica DFC 420C camera. R packages were used for statistical analysis.

### RNAseq and differential expression analysis

Mycelia of the *Δtmk1*, *Δtmk3* and WT strains were collected 30 min following mycelial damage and frozen immediately. The WT strain was previously treated with 10 mM BAPTA or 100 μM ATP for 15 min, as indicated. In all cases, an injured control without chemical treatment and a control without injury were included, and three biological replicates were analyzed per strain and/or treatment. Total RNA was extracted with TRIzol (Invitrogen).

Libraries for RNAseq were prepared using the TruSeq RNA library preparation protocol (Illumina). Each library was sequenced using a NextSeq500 sequencer in the 1x75 format. The 75-bp reads were pseudo-aligned to the *T*. *atroviride* V2 transcripts, using *kallisto* [68]. On average, 25 million reads per library were obtained with high quality (S2 Table). The RNAseq data discussed in this publication have been deposited in NCBI’s Gene Expression Omnibus [69] and are accessible through GEO Series accession number GSE115811 (https://www.ncbi.nlm.nih.gov/geo/query/acc.cgi?acc=GSE115811).

For the differential gene expression analysis, only those genes that had at least three counts per million in at least ten libraries were considered as transcribed. All analyses were carried out using the *edgeR* package [70]. For determining differential expression between the comparisons, we used the generalized linear model (GLM) likelihood ratio test. False discovery rates (FDR) were calculated and genes with an FDR < 0.05 and absolute log_2_Fold-change ≥ 1 were considered differentially expressed. Venn diagrams and heatmaps were constructed to compare the universes of differentially expressed genes using the *gplots* package in R. To identify the set of genes related to regeneration, a highly restrictive differential expression analysis was performed with a value of FDR < 0.01. A Fold-change cutoff was not considered in this case.

Enrichment analyses for Cellular Component and Biological Process GO terms were performed using camera, from the *edgeR* package [71]. GO terms with FDR ≤ 0.05 were considered significantly enriched in each comparison. We have presented this data in a clustered heatmap that highlights the categories enriched with asterisks, where ** represents FDR < 0.01 and * FDR < 0.05. The plotted values are the percentage of genes belonging to each category that are deemed differentially expressed (see previous section). The GO terms were first filtered for redundancy, removing those that contained more than 1000 or less than 4 genes.

### Quantitative RT-PCR

To validate the differential expression of regeneration associated genes, primers for qRT-PCR were designed to produce amplicons around 150 bp (S1 Table). cDNA was synthesized using as template RNA extracted from injured and control mycelia, and RT II SuperScript (Invitrogen) using four biological and three technical replicates. The reaction mixture for quantitative PCR was as follows: 10 μl of SYBR green master mix (Applied Biosystems), 3 μl of cDNA template (3ng/μl) and 1 μl of each (10 μM) of the primers. The PCR program was as follows: One cycle at 95°C for 5 min, 40 cycles at 95°C each for 30 s, at 65°C for 30 s, 72°C for 40 s. Melting curves for each product, starting from 60°C to 95°C at 0.2°C/s, produced a single melting point. All qRT-PCR reactions were repeated three times. Anova and Tuckey tests were performed to determine the significance of changes in gene expression.

## Supporting information

S1 Fig Comparative analysis of effect of ATP and injury in gene enrichment. Clustering of enriched Biological Process Gene Ontology terms in response to injury and ATP addition, showing the log_10_ of the FDR value (FDR <0.01**; FDR <0.05*).
(TIF)

S2 Fig Gene enrichment analysis. Biological Process GO enrichment analysis of the intersection between the up-regulated genes in response to injury in the WT strain and those up-regulated in the *“Regeneration vs No-Regeneration”* comparison. The result of removing redundant GO terms is shown in a semantic similarity-based network, performed with REVIGO.
(TIF)

S1 Table List of primers used in this work. The table shows the sequences of all oligonucleotides used in this work.
(DOCX)

S2 Table Sequencing statistics. The table shows the sequencing and alignment statistics for each of the RNAseq library used in this work.
(DOCX)

S1 Movie Live cell imaging. The *T*. *atroviride* WT strain carrying pEM12 was damaged with a scalpel and visualized using a confocal laser scanning microscope fitted with an argon/2 ion laser (EGFP: excitation, 488 nm; emission, 510 nm). Image projections consisting of stacks of images were captured during a 5 min period and converted into a movie using a confocal microscope.
(MOV)

S2 Movie Live cell imaging. The undamaged *T*. *atroviride* WT strain carrying pEM12 was visualized using a confocal laser scanning microscope fitted with an argon/2 ion laser (EGFP: excitation, 488 nm; emission, 510 nm). Image projections consisting of stacks of images were captured during a 5 min period and converted into a movie using a confocal microscope.
(MOV)

S1 Dataset Annotation of genes of differentially expressed genes upon BAPTA treatment. The table is a list of genes of the WT strain exclusively responsive to injury in absence of BAPTA, those exclusive to injury in the presence of BAPTA, and those responsive in the two treatments.
(XLSX)

S2 Dataset Deferentially expressed genes in the comparison WT+BAPTA vs WT-Control. The table includes data on expression level and statistical support summary for expression differences.
(XLSX)

S3 Dataset Deferentially expressed genes in the comparison WT+BAPTA vs WT-Injury. The table includes data on expression level and statistical support summary for expression differences.
(XLSX)

S4 Dataset Deferentially expressed genes in the comparison WT-Injury vs WT-Control. The table includes data on expression level and statistical support summary for expression differences.
(XLSX)

S5 Dataset Deferentially expressed genes in the comparison WT-eATP vs WT-Control. The table includes data on expression level and statistical support summary for expression differences.
(XLSX)

S6 Dataset Deferentially expressed genes in the comparison tmk1-Injury vs tmk1-Control. The table includes data on expression level and statistical support summary for expression differences.
(XLSX)

S7 Dataset Deferentially expressed genes in the comparison tmk3-Injury vs tmk3-Control. The table includes data on expression level and statistical support summary for expression differences.
(XLSX)

S8 Dataset Annotation of genes of the comparison of the transcriptional response to injury of the Δ*tmk* mutants. The table is a list of genes responsive to injury exclusively in each of the strains analyzed (WT, Δ*tmk*1, Δ*tmk*3) and those responsive in the WT and each of the mutants.
(XLSX)

S9 Dataset Up-regulated Regeneration Associated Gene Set. The table lists the 9 groups of genes up-regulated during regeneration classified according to their manually curated functional annotation.
(XLSX)

S10 Dataset Down regulated Regeneration Associated Gene Set. The table shows a complete list of genes down-regulated during regeneration and their functional annotation.
(XLSX)
