# Supplementary figures and images for: Danger signals activate a putative innate immune system during regeneration in a filamentous fungus
Source: PLoS Genet. 2018 Nov 30;14(11):e1007390. doi: 10.1371/journal.pgen.1007390 (PMC6291166; doi:10.1371/journal.pgen.1007390)

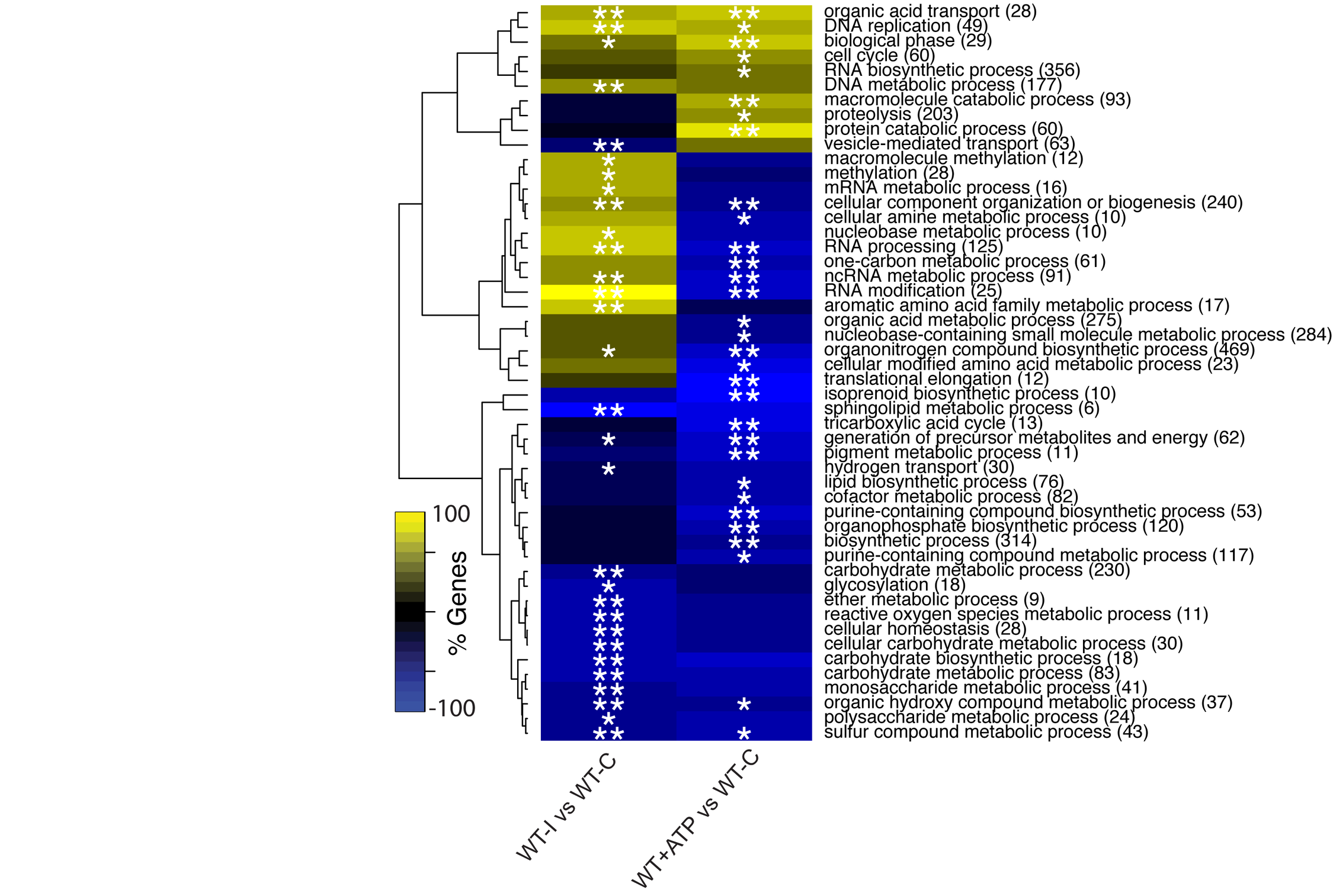

Supplement: S1 Fig — Clustering of enriched Biological Process Gene Ontology terms in response to injury and ATP addition, showing the log10 of the FDR value (FDR <0.01**; FDR <0.05*). (TIF) [file pgen.1007390.s001.tif]

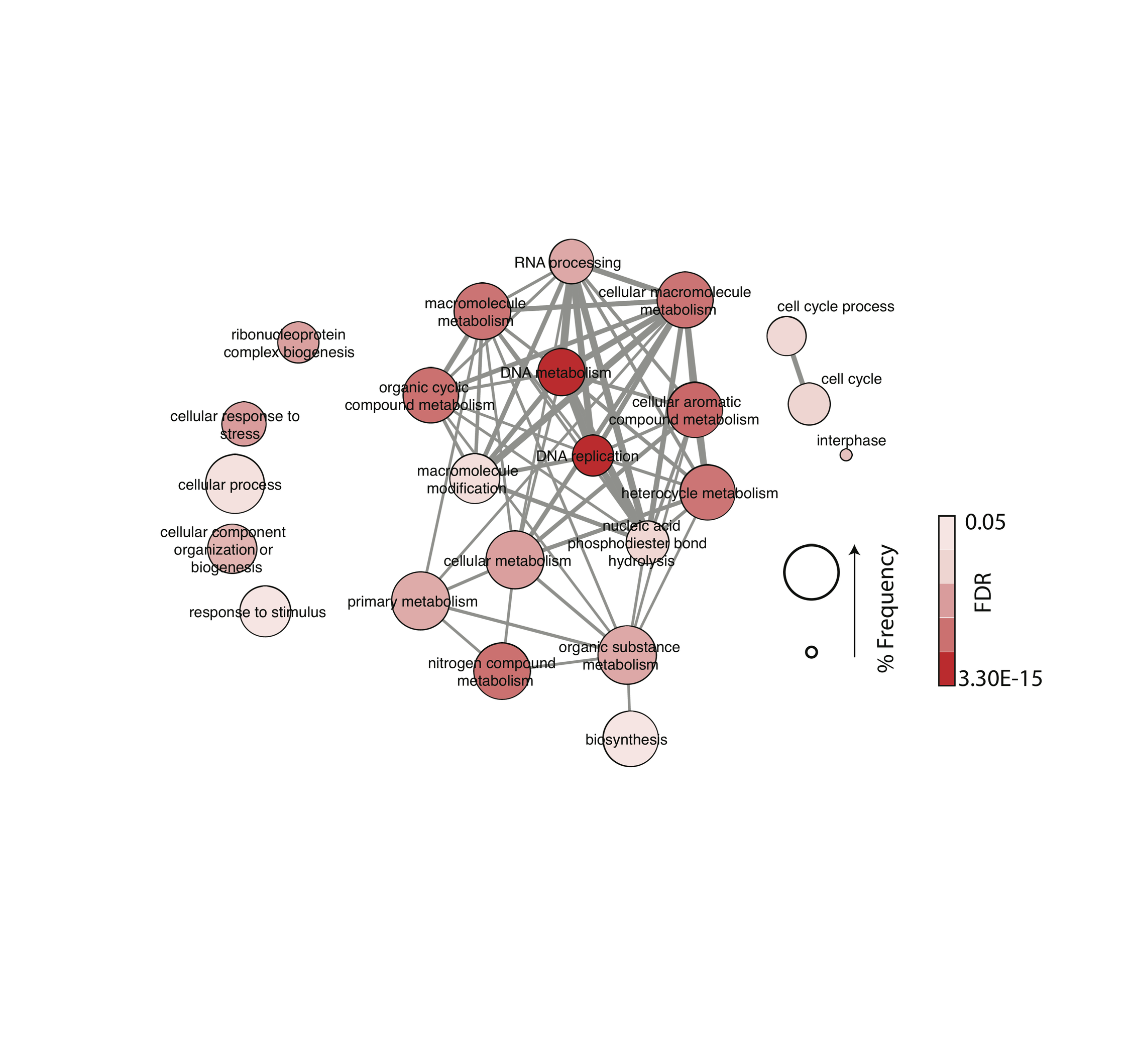

Supplement: S2 Fig — Biological Process GO enrichment analysis of the intersection between the up-regulated genes in response to injury in the WT strain and those up-regulated in the “Regeneration vs No-Regeneration” comparison. The result of removing redundant GO terms is shown in a semantic similarity-based network, performed with REVIGO. (TIF) [file pgen.1007390.s002.tif]
