# Supplementary material for: Danger signals activate a putative innate immune system during regeneration in a filamentous fungus
Source: PLoS Genet. 2018 Nov 30;14(11):e1007390. doi: 10.1371/journal.pgen.1007390 (PMC6291166; doi:10.1371/journal.pgen.1007390)
Supplement: S1 Table — The table shows the sequences of all oligonucleotides used in this work. (DOCX) [file pgen.1007390.s003.docx]

Table S1. Primers used in this work. The table shows the sequences of all oligonucleotides used in this work and their use.

| **Name** | **Sequence** | **Use** |
| --- | --- | --- |
| GCaMP6-EcoRI-FW | CGGGCTGCAGGAATTCATGGGTTCTCATC  ATCATCATCATCATG | Amplification of the ORF GCaMP6 |
| GCaMP6-SalI-RV | GGCCCTCGAGGTCGACTCACTTCGCTGTC  ATCATTTGTACAAAC | Amplification of the ORF GCaMP6 |
| TBlu17-RV | GGGGCAAAGACAATTGATAGAACAGCA | Diagnostic PCR of pEM12 |
| GCaMP6-FW | ATGGGTTCTCATCATCATCATCATCATG | Diagnostic PCR of pEM12 |
| GCaMP6-RV | TCACTTCGCTGTCATCATTTGTACAAAC | Diagnostic PCR of pEM12 |
| Nacht prot FW | TTTGAATCAGAGCGTGCTATCGCG | qRT-PCR |
| Nacht prot RV | CCTCCAGAAATTGGTAAATGCGC | qRT-PCR |
| Het-s294334 FW | CGTCTATGGCTTGGATGTTGCC | qRT-PCR |
| Het-s294334 RV | GTCACAGGTGCGCGAAGCC | qRT-PCR |
| CMK1 FW | AAATCGAGGCCTTCCGCAGACG | qRT-PCR |
| CMK1 RV | CGTCTCCCATTTCCGAGTTCTCG | qRT-PCR |
| Rad5 FW | AGCAGATGAGATGGGGCTGGG | qRT-PCR |
| Rad5 RV | TTGTCCGGGATTGTCTGGGTAGC | qRT-PCR |
| DNA pol FW | GAGTGGCGTATGTCATGGTG | qRT-PCR |
| DNA pol RV | GCGTCTTCTTGGCAAACTTC | qRT-PCR |
