# Supplementary material for: Danger signals activate a putative innate immune system during regeneration in a filamentous fungus
Source: PLoS Genet. 2018 Nov 30;14(11):e1007390. doi: 10.1371/journal.pgen.1007390 (PMC6291166; doi:10.1371/journal.pgen.1007390)
Supplement: S2 Table — The table shows the sequencing and alignment statistics for each of the RNAseq library used in this work. (DOCX) [file pgen.1007390.s004.docx]

Table S2. Sequencing statistics. The table shows the sequencing and alignment statistics for each of the RNAseq library used in this work.

| ***Library*** | ***Number of reads*** | ***Reads pseudoaligned*** | ***% pseudoaligned*** | **% GC** | **Mean Quality** |
| --- | --- | --- | --- | --- | --- |
| **ATP_R1** | 29,915,377 | 23,553,436 | 78.73 | 54 | 34 |
| **ATP_R2** | 32,748,259 | 26,476,053 | 80.85 | 53 | 34 |
| **ATP_R3** | 25,354,828 | 19,267,967 | 75.99 | 54 | 34 |
| **tmk1_C_R1** | 27,430,440 | 21,821,496 | 79.55 | 53 | 34 |
| **tmk1_C_R2** | 36,831,150 | 29,565,784 | 80.27 | 54 | 34 |
| **tmk1_C_R3** | 38,303,657 | 30,264,214 | 79.01 | 54 | 34 |
| **tmk1_I_R1** | 13,282,762 | 10,741,585 | 80.87 | 54 | 34 |
| **tmk1_I_R2** | 35,254,942 | 27,317,502 | 77.49 | 54 | 34 |
| **tmk1_I_R3** | 18,178,452 | 14,372,412 | 79.06 | 54 | 34 |
| **tmk3_C_R1** | 20,352,669 | 16,693,801 | 82.02 | 52 | 34 |
| **tmk3_C_R2** | 20,010,487 | 15,438,510 | 77.15 | 53 | 34 |
| **tmk3_C_R3** | 28,735,516 | 21,991,049 | 76.53 | 54 | 34 |
| **tmk3_I_R1** | 24,410,144 | 18,505,063 | 75.81 | 53 | 34 |
| **tmk3_I_R2** | 23,300,246 | 17,809,274 | 76.43 | 53 | 34 |
| **tmk3_I_R3** | 24,245,580 | 18,590,130 | 76.67 | 53 | 34 |
| **WT_bapta_R1** | 21,150,780 | 16,742,058 | 79.16 | 54 | 34 |
| **WT_bapta_R2** | 21,187,446 | 16,565,942 | 78.19 | 54 | 34 |
| **WT_bapta_R3** | 30,519,083 | 23,729,779 | 77.75 | 54 | 34 |
| **WT_C_R1** | 21,770,455 | 18,073,692 | 83.02 | 52 | 34 |
| **WT_C_R2** | 15,586,542 | 11,974,338 | 76.82 | 53 | 34 |
| **WT_C_R3** | 15,799,760 | 12,122,701 | 76.73 | 53 | 34 |
| **WT_I_R1** | 27,464,653 | 20,747,559 | 75.54 | 54 | 34 |
| **WT_I_R2** | 30,713,438 | 24,060,680 | 78.34 | 54 | 34 |
| **WT_I_R3** | 26,074,913 | 20,351,200 | 78.05 | 54 | 34 |
| **Total** | **608,621,579** | **476,776,225** |  |  |  |
| **Average** | 25,359,232 | 19,865,676 |  |  |  |
